# Development of a highly sensitive voltammetric sensor for the detection of folic acid by using MoS_2_ and ionic liquid-modified carbon paste electrode

**DOI:** 10.5599/admet.1823

**Published:** 2023-07-22

**Authors:** Hadi Soltani Nejad, Fariba Garkani Nejad, Hadi Beitollahi

**Affiliations:** 1 Department of Chemistry, Graduate University of Advanced Technology, Kerman, Iran; 2 Environment Department, Institute of Science and High Technology and Environmental Sciences, Graduate University of Advanced Technology, Kerman 7631818356, Iran

**Keywords:** vitamin B_9_, transition metal dichalcogenides, electrocatalysis

## Abstract

**Background and purpose:**

Sensitive analytical determination of folic acid is important in clinical laboratories due to its versatile biological functions.

**Experimental approach:**

A simple folic acid sensor was successfully fabricated based on two-dimensional transition metal dichalcogenide MoS_2_ modified carbon ionic liquid paste electrode (MoS_2_-CILPE). The electrochemical properties of the fabricated electrode were investigated by cyclic voltammetry (CV), differential pulse voltammetry (DPV), and chronoamperometry.

**Key results:**

The fabricated sensor displayed excellent electroactivity towards folic acid using CV. Under optimal conditions (0.1 M PBS (pH 7.0)), the DPV oxidation peak current was proportional to folic acid concentration in the range from 5.0 μM to 100.0 μM with an estimated limit of detection of 1.0 μM and limit of quantification of 5.0 μM.

**Conclusion:**

The ability of the sensor for routine analyses was demonstrated by the detection of folic acid present in folic acid tablets and urine samples with appreciable recovery values.

## Introduction

Recently, significant progress has been made in the development of electrochemical sensors and their application in point-of-care diagnostics, environmental studies, food safety, drug screening, and security [1-3]. Low cost, simplicity, high reproducibility, real-time measurements, rapid response, low detection limit and portable devices are some advantages that cause extensive interest in electrochemical methods. Particularly, voltammetric techniques are extremely sensitive and selective for the detection of easily oxidizable analytes [4-7]. To achieve sensitivity and selectivity, the modification of the working electrode in voltammetry is a usual practice. These chemically modified electrodes gain considerable attention in electrochemical quantification studies due to the enhanced electron transfer rate as well as selectivity achieved due to modifications [8-11].

Carbon paste electrode (CPE) has been widely used in the determination of drugs, vitamins and other species because of its specific properties like easy preparation and wider potential window. The modifier has an important effect on the performance of modified CPEs for electrochemical measurement [12-15].

Recent activity has focused on the development of nanoscaled particles applied in analytical chemistry to obtain special physicochemical characteristics of electrodes [16-21]. For example, nanomaterials with a large surface area, good conductivity, and excellent biological compatibility can be used as signal amplification elements in electrochemical sensors. Therefore, exploring new advanced nanomaterials is key to developing sensors with a high sensitivity and low detection limit [22-24].

2D layered nanomaterials are an emerging but important class of materials. They refer to materials with one dimension restricted to a single-atom layer, including monolayer and few-layer nanomaterials [25-27]. In the 2D family, layered transition metal dichalcogenides (TMDs), such as MoS_2_, WS_2_, TiS_2_, TaS_2_, MoSe_2_, and WSe_2_, are fundamentally and technologically intriguing [28].

Layer-structured transition-metal dichalcogenides possess an unique layered structure of similar structure to graphite and large surface areas, as well as outstanding physical, chemical, optical, and electronic properties, which holds great potential for applications in catalysis, sensing, optics, and energy [29-31]. Because of its ultra thin layer structure, specific electrochemical properties, band gap (t1.9 eV), large active edges, and easy surface modification, MoS_2_ becomes one of the fascinating candidates to construct electrochemical sensors with high performance. As one of the layer-structured transition-metal dichalcogenides, MoS_2_ has an analogous structure to graphite, which is composed of three atom layers: a Mo layer sandwiched between two S layers, and the triple layers are stacked and held together by weak van der Waals interactions.

There are recent reports on using ionic liquids to design high sensitive electrochemical sensors. Ionic liquids possess high ionic conductivity, high chemical and thermal stabilities, and high viscosity, and they are promising candidate materials for the fabrication of electrochemical sensors [32-34].

Folic acid (FA), (2S)-2-[(4-{[(2-amino-4-hydroxypteridin-6-yl) methyl]amino}phenyl)formamido]pentanedioic acid, also known as folate (the natural form in body), vitamin B_9_, vitamin Bc (or folacin), pteroyl-L-glutamic acid, pteroyl-L-glutamate and pteroylmonoglutamic acid are essential for numerous bodily functions. Since humans cannot synthesize folate, the consumption of natural sources such as some green-leafy vegetables or fortified food and tablets is necessary [35-38]. Several chronic diseases, for example, gigantocytic anemia, leucopoenia, mentality devolution, psychosis, heart attack, and stroke, are related to the deficiency of FA. It has also been suggested that decreased folate concentration is associated with enhanced carcinogenesis as folic acid with vitamin B_12_ participates in the nucleotide synthesis, cell division and gene expression. Besides, it is an essential nutrient for pregnant women to prevent neural tube defects in the fetus [39-42]. So, a sensitive determination of FA from a clinical viewpoint is very important.

In this study, MoS_2_ modified carbon ionic liquid paste electrode (MoS_2_-CILPE) sensor was fabricated as a highly sensitive voltammetric sensor to determine the FA. The MoS_2_-CILPE sensor showed an acceptable ability to determine the folic acid in folic acid tablets and urine samples.

## Experimental

### Chemicals and instrumentation

All chemicals used were of analytical grade and were used as received without any further purification and were obtained from Sigma-Aldrich. Orthophosphoric acid was utilized to prepare the phosphate buffer solutions (PBSs), and sodium hydroxide was used to adjust the desired pH values (pH range between 2.0 and 9.0).

All solutions were prepared with deionised water of Millipore Direct-Q^®^ 8 UV (ultra-violet) (Millipore, Germany). The pH was also measured and a buffer solution was prepared using a digital pH meter (Metrohm, pH Lab 713). Voltammetric measurements were carried out using an Autolab PGSTAT302N, potentiostat/galvanostat (made in Netherlands). The system was run on a PC using General Purpose Electrochemical System (GPES) 4.9 software. A three-electrode system was used, including a platinum wire as the auxiliary electrode, an Ag/AgCl/KCl (saturated) as the reference electrode, and the MoS_2_-CILPE as the working electrode. The synthesis and characterization of 2D MoS_2_ nanosheets has been reported in our previous work [43].

### Preparation of MoS_2_-CILPE

MoS_2_-CILPE was prepared by mixing 0.04 g of MoS_2_ nanosheets with 0.96 g graphite powder and the appropriate amount of ionic liquid (1-butyl-3-methylimidazolium hexafluorophosphate) and paraffin oil (30/70 (w/w)) with a mortar and pestle. The paste was then packed into the end of a glass tube (3.4 mm inner diameter and 15 cm long). A copper wire inserted into the carbon paste provided the electrical contact. For comparison, carbon ionic liquids paste electrode (CILPE) in the absence of MoS_2_ nanosheets, MoS_2_-CPE consistent of MoS_2_ nanosheets powder, graphite powder and paraffin oil, and bare CPE consisting of graphite powder and paraffin oil were also prepared in the same way.

### Preparation of real samples

Five tablets of the FA purchased from a local pharmacy in Kerman, Iran (1 mg FA per tablet) were completely powdered in the mortar with a pestle. Then, an accurately weighed amount of the homogenized FA powder was transferred into 100 mL 0.1 M PBS (pH 7.0). For better dissolution, the solutions inside the flasks were sonicated (20 min). After that, the resulting samples were filtered. Finally, a specific volume of the prepared samples was transferred to volumetric flasks and diluted with 0.1 M PBS (pH 7.0). The diluted solutions were then put in the electrochemical cell for DPV analysis.

The collected urine samples were stored in the refrigerator after collection. The urine sample was centrifuged for 5 minutes at 2000 rpm. Then, the supernatant solution was filtered after phase separation and diluted with 0.1 M PBS (pH 7.0). The diluted solution was then put in the electrochemical cell for DPV analysis. The analytical experiments were performed using the standard addition method.

## Results and discussion

### Electrochemical behavior of FA on the MoS_2_-CILPE

**Figure 1.**
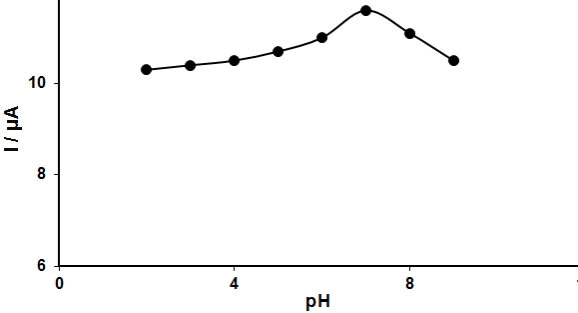
Plot of the oxidation peak current of 100.0 μM FA as a function of pH solution at MoS_2_-CILPE in 0.1 M PBS at different pH value (2.0-9.0).

Mechanism of the FA oxidation on MoS_2_-CILPE is suggested on the basis of the relationship between the oxidation potential and pH of supporting electrolyte. The effect of the electrolyte pH on the oxidation of 100.0 μM FA was investigated at MoS_2_-CILPE using DPV measurements in the PBS in the pH range from 2.0 to 9.0. According to the results, the oxidation peak current of FA depends on the pH value. It increases with increasing pH until it reaches the maximum at pH 7.0, then decreases with higher pH values. The optimized pH corresponding to the higher peak current was 7.0 (Fig. 1), indicating that protons are involved in the reaction of FA oxidation (2 electrons and 2 protons).

**Figure 2.**
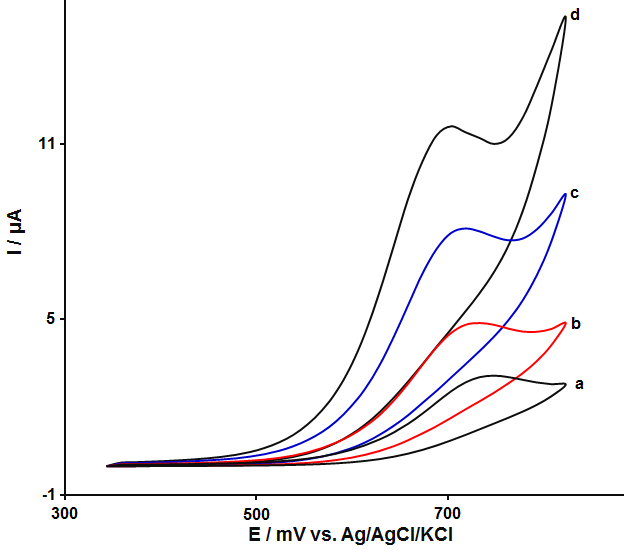
Cyclic voltammetric response of 100.0 μM folic acid at (a) bare CPE, (b) MoS_2_-CPE, (c) IL-CPE and d) MoS_2_-CILPE in 0.1 M PBS of pH 7.0 (Scan rate = 50 mV s^1^) in the potential window of 350-820 mV.

The effect of MoS_2_ nanosheets and ILs in the modification process was investigated by recording cyclic voltammograms of 100.0 μM FA at the surface of CPE (curve a), MoS_2_-CPE (curve b), ionic liquid modified carbon paste electrode (IL-CPE) (curve c) and MoS_2_-CILPE (curve d). The results are shown in Figure 2. The oxidation current and potential for FA were detected at about 3.1 μA and 750 mV at the surface of CPE and 4.8 μA and 732 mV at the surface of MoS_2_-CPE, respectively. On IL-CPE, the oxidation peak was located at 725 mV with an oxidation peak height of 8.0 μA (curve c). It can be seen that the oxidation peak potential moved to the negative direction with a significant increase of the oxidation peak current attributed to the presence of ionic liquid as the modifier in the carbon paste electrode. The modification of CPE with MoS_2_ nanosheets and ILs improved the oxidation current of FA (11.7 μA) and decreased the oxidation potential of FA (700 mV) compared with the bare CPE.

### Effect of scan rate

**Figure 3.**
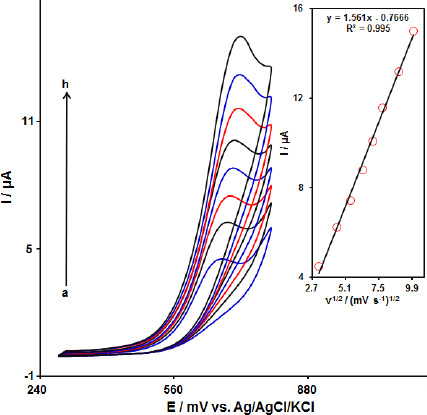
Cyclic voltammetric responses of 90.0 μM FA in 0.1 M PBS (pH 7.0) at scan rates of 10 to 100 mV s^-1^ at MoS_2_-CILPE (a-h refers to 10, 20, 30, 40, 50, 60, 80, and 100 mV s^-1^) in the potential window of 280-800 mV. Inset: Plot of the square root of the scan rate vs. the oxidation peak current of FA.

The effect of the potential scan rates (10-100 mV s^-1^) on the electrochemical oxidation of FA was studied by CV. Figure 3 shows the CV of 90.0 μM of FA in the 0.1 M PBS at the MoS_2_-CILPE. These results show that the anodic current increased with an increasing scan rate. The oxidation current of FA increased linearly with the square root of the scan rate (Figure 3, Inset), demonstrating a diffusion-controlled electrochemical process.

### Chronoamperometric analysis

**Figure 4.**
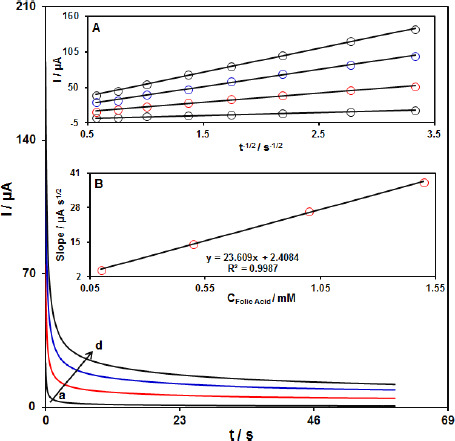
The chronoamperograms obtained at MoS_2_-CILPE in 0.1 M PBS at pH 7.0 for different concentrations of FA at step potential = 750 mV. Noted that a–d related to 0.1, 0.5, 1.0, and 1.5 mM of FA. Inset A: The *I* plot versus *t*^-1/2^ observed by chronoamperograms a to d. Inset B: Slope plot of the straight line vs. concentration of FA.

The chronoamperometric measurements of FA at the MoS_2_-CILPE surface were done to estimate the apparent diffusion coefficient. Figure 4 shows the current-time profiles obtained by setting the working electrode potential at 750 mV for different concentrations of FA.

At long enough experimental times (*t* = 0.3 to 3 s), where the electron transfer reaction rate of FA is more than its diffusion rate toward the working electrode surface, the current is diffusion controlled. Figure 4, inset A, shows the experimental plots of *I* versus *t*^-1/2^ with the best fit for different concentrations of FA employed. The slopes of the resulting straight lines were then plotted versus the FA concentration (Figure 4, inset B). Based on the Cottrell equation (The Cottrell equation is *I* = *nFAC* (*D*/π*t*)^1/2^, where *D* is the diffusion coefficient (cm^2^ s^-1^), *C* is the concentration in bulk solution (mM), *A* is the surface area of the electrode (cm^2^), *F* is Faraday’s constant, *t* is the time (s), and *n* is the number of electrons transferred), the slope of this plot (Figure 4 inset B) can be used to estimate of the diffusion coefficient of FA. From the slope of this plot, the value of *D* was found to be 5.7×10^-6^ cm^2^s^-1^ for FA.

### Calibration plot and limit of detection

**Figure 5.**
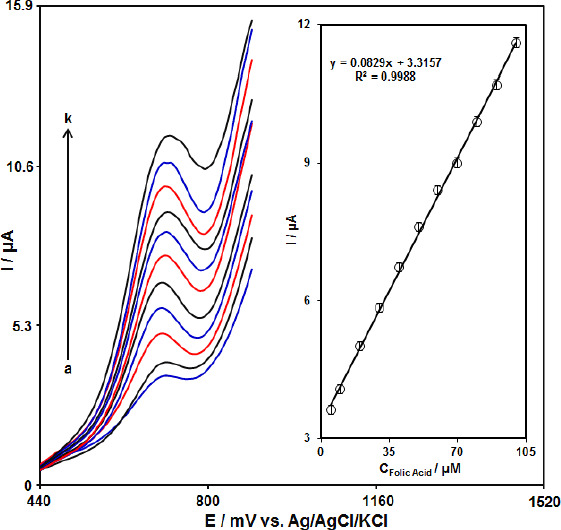
DPV response of FA at MoS_2_-CILPE in the concentration range 5.0 to 100.0 μM in 0.1 M PBS of pH 7.0 (a-k refers to 5.0, 10.0, 20.0, 30.0, 40.0, 50.0, 60.0, 70.0, 80.0, 90.0, and 100.0 μM). Inset: the peak current plot as a function of the FA concentration ranging from 5.0 to 100.0 μM.

Since DPV has a much higher current sensitivity and better resolution than CV, DPV was used for the determination of FA. Figure 5 shows the DPV curves of MoS_2_-CILPE in the PBS with variable FA levels (Step potential = 0.01 V and pulse amplitude =0 .025 V). It was found that the electrocatalytic peak currents of FA oxidation at the MoS_2_-CILPE surface linearly depended on FA concentrations above the range of 5.0 to 100.0 μM. The limit of detection is estimated by using the following equation, LOD = 3*S*_b_/*m*. In this equation, *m* is the slope of the calibration plot (0.0829 μA μM^-1^) and *S*_b_ is the standard deviation of the blank response, obtained from 8 replicate measurements of the blank solution. The limit of detection was 1.0 μM and the limit of quantification (LOQ) was obtained 5.0 μM. A comparison of FA detection using various sensors is presented in Table 1.

### Interference studies

To evaluate the selectivity of MoS_2_-CILPE for FA, an investigation of the influence of potential interfering substances was performed under the optimized conditions. The DPV responses after adding interfering substances into 0.1 M PBS (pH 7.0) containing 50.0 μM FA were recorded. The tolerance limit was defined as the ratio of the concentration of the interfering species to the analyte, which led to a relative error of less than ±5.0 %. It was found that the 500-fold excess of glucose, glycine, methionine, histidine, alanine, glutamic acid, glycine, phenylalanine, 400-fold excess of Ca^2+^, Na^+^, Mg^2+^, NH_4_^+^, Cl^-^, SO_4_^2-^, 70-fold excess of urea, uric acid, and 10-fold of ascorbic acid did not remarkable interfere for FA determination.

### Stability, reproducibility, and repeatability of the MoS_2_-CILPE sensor

For evaluation of the reproducibility of the prepared sensor, five MoS_2_-CILPE were prepared independently and used in the determination of FA through DPV in PBS (0.1 M, pH 7.0). Under the same experimental conditions, the calculated relative standard deviation (RSD) of peak currents was only about 4.1 %, indicating reliable reproducibility of the sensing platform.

The storage stability of the MoS_2_-CILPE was further examined by measurement of the FA oxidation peak current over the time interval of 12 days. No obvious decrease in the initial current value of FA was observed after 12 days, implying acceptable storage stability.

The repeatability of the MoS_2_-CILPE sensor was studied by 5 consecutive measurements of 50.0 μM FA with RSD of 3.5 %, indicating good repeatability of the sensor.

### Analysis of real samples

The real samples for the analysis were prepared and quantified by the DPV method. The developed sensor was applied to detect FA in folic acid tablets and urine samples. The results are summarized in Table 2. Each measurement was repeated five times. The FA acid content of each tablet was obtained at 1.003 mg. The recovery and relative standard deviation (RSD) values confirmed that the MoS_2_-CILPE sensor has great potential for analytical application.

## Conclusion

A sensitive and reliable electrochemical method based on MoS_2_-CILPE was proposed for the determination of FA. Due to the large surface area of MoS_2_, high conductivity and catalytic activity of ionic liquid, the modified electrode exhibited good catalytic activity to FA with enhanced oxidation peak current and decreased oxidation overpotential. The voltammetric current response increased linearly with increasing FA concentration in the range of 5.0 to 100.0 μM and the detection limit of 1.0 μM was obtained. Moreover, the MoS_2_-CILPE sensor may provide a facile and effective analysis approach for the determination of FA in real samples.

## Figures and Tables

**Table 1.** Comparison of different DPV sensors for FA detection,.

Electrochemical sensor	Linear range, μM	LOD, μM	Ref.
MoS_2_-reduced graphene oxide hybrid/glassy carbon electrode	0.01-100	0.010	[44]
Ferrocene dicarboxylic acid/carbon nanotube paste	4-152	1.1	[45]
Methylene blue-reduced graphene oxide/glassy carbon electrode	4-167	0.500	[46]
ZrO_2_ nanoparticles-carbon paste electrode	20–2500	9.86	[47]
Multi-walled carbon nanotubes-Pt nanoparticles/glassy carbon electrode	0.2-100	0.050	[48]
MoS_2_-CILPE	5.0-100.0	1.0	This work

**Table 2.** Determining FA in folic acid tablets and urine through MoS_2_-CILPE. All the concentrations are in μM (n = 5).

Sample	FA concentration, μM		Recovery, %
	Spiked	Found	
Folic Acid Tablet	0	3.5±0.01	-
	1.0	4.4±0.015	97.8
	2.0	5.6±0.02	101.8
	3.0	6.7±0.012	103.1
	4.0	7.4±0.01	98.7
Urine	0	-	-
	5.0	4.9±0.011	98.0
	6.0	6.1±0.016	101.7
	7.0	6.8±0.013	97.1
	8.0	8.3±0.019	103.7
